# Particle Beam Radiobiology Status and Challenges: A PTCOG Radiobiology Subcommittee Report

**DOI:** 10.1016/j.ijpt.2024.100626

**Published:** 2024-08-08

**Authors:** Reem Ahmad, Amelia Barcellini, Kilian Baumann, Malte Benje, Tamara Bender, Paloma Bragado, Alexandra Charalampopoulou, Reema Chowdhury, Anthony J. Davis, Daniel K. Ebner, John Eley, Jake A. Kloeber, Robert W. Mutter, Thomas Friedrich, Alvaro Gutierrez-Uzquiza, Alexander Helm, Marta Ibáñez-Moragues, Lorea Iturri, Jeannette Jansen, Miguel Ángel Morcillo, Daniel Puerta, Anggraeini Puspitasari Kokko, Daniel Sánchez-Parcerisa, Emanuele Scifoni, Takashi Shimokawa, Olga Sokol, Michael D. Story, Juliette Thariat, Walter Tinganelli, Francesco Tommasino, Charlot Vandevoorde, Cläre von Neubeck

**Affiliations:** 1 Department of Medical Physics and Biomedical Engineering, University College London, London, UK; 2 Department of Internal Medicine and Therapeutics, University of Pavia, Pavia, Italy; 3 Clinical Department Radiation Oncology Unit, National Center for Oncological Hadrontherapy (CNAO), Pavia, Italy; 4 Institute of Medical Physics and Radiation Protection, University of Applied Sciences Giessen, Giessen, Germany; 5 Marburg Ion-Beam Therapy Center, Marburg, Germany; 6 Biophysics Department, GSI Helmholtzzentrum für Schwerionenforschung, Darmstadt, Germany; 7 Biochemistry and Molecular Biology Department, Complutense University of Madrid, Madrid, Spain; 8 University School for Advanced Studies (IUSS), Pavia, Italy; 9 Radiobiology Unit, Development and Research Department, National Center for Oncological Hadrontherapy (CNAO), Pavia, Italy; 10 University of Texas Southwestern Medical Center, Dallas, Texas, USA; 11 Department of Radiation Oncology, Mayo Clinic, Rochester, Minnesota, USA; 12 Department of Radiation Oncology, Vanderbilt University School of Medicine, Nashville, TN 37232, USA; 13 Medical Applications of Ionizing Radiation Unit, Technology Department, Centro de Investigaciones Energéticas, Medioambientales y Tecnológicas (CIEMAT), Madrid, Spain; 14 Institut Curie, Université PSL, CNRS UMR3347, Inserm U1021, Signalisation Radiobiologie et Cancer, Orsay, France; 15 Departamento de Física Atómica, Molecular y Nuclear, Universidad de Granada, Granada, Spain; 16 Instituto de Investigación Biosanitaria (ibs.GRANADA), Complejo Hospitalario Universitario de Granada/Universidad de Granada, Granada, Spain; 17 HollandPTC, Delft, the Netherlands; 18 Gunma University Heavy Ion Medical Center, Maebashi, Japan; 19 Grupo de Física Nuclear, IPARCOS & IdISSC, Universidad Complutense de Madrid, Madrid, Spain; 20 TIFPA-INFN - Trento Institute for Fundamental Physics and Applications, Trento, Italy; 21 National Institutes for Quantum Science and Technology (QST), Chiba, Japan; 22 Mayo Clinic, Jacksonville, Florida, USA; 23 Centre François Baclesse, Université de Caen Normandie, ENSICAEN, CNRS/IN2P3, LPC Caen UMR6534, Caen, France; 24 Department of Physics, University of Trento, Trento, Italy; 25 Department of Particle Therapy, University Hospital Essen, University of Duisburg-Essen, Duisburg, Germany

**Keywords:** Particle therapy, Bragg peak, Radiobiology

## Abstract

Particle therapy (PT) represents a significant advancement in cancer treatment, precisely targeting tumor cells while sparing surrounding healthy tissues thanks to the unique depth-dose profiles of the charged particles. Furthermore, their linear energy transfer and relative biological effectiveness enhance their capability to treat radioresistant tumors, including hypoxic ones. Over the years, extensive research has paved the way for PT's clinical application, and current efforts aim to refine its efficacy and precision, minimizing the toxicities. In this regard, radiobiology research is evolving toward integrating biotechnology to advance drug discovery and radiation therapy optimization. This shift from basic radiobiology to understanding the molecular mechanisms of PT aims to expand the therapeutic window through innovative dose delivery regimens and combined therapy approaches. This review, written by over 30 contributors from various countries, provides a comprehensive look at key research areas and new developments in PT radiobiology, emphasizing the innovations and techniques transforming the field, ranging from the radiobiology of new irradiation modalities to multimodal radiation therapy and modeling efforts. We highlight both advancements and knowledge gaps, with the aim of improving the understanding and application of PT in oncology.

## Introduction

Particle therapy (PT) has emerged as a revolutionary tool in cancer treatment, offering precision in targeting tumor cells while minimizing damage to surrounding healthy tissues. Originating from the concept of Robert R. Wilson at Lawrence Berkeley National Laboratory, the primary objective has always been to harness the unique properties of ions to optimize radiation dose delivery.^1^, ^2^ Charged particles exhibit a characteristic dose distribution (Bragg peak) depositing most of their energy at the end of their track. This energy deposition can be directed to the tumor site, minimizing damage to critical organs beyond it.^2^, ^3^ Apart from the favorable dose distribution, the relative biological effectiveness (RBE) of PT is larger than that of x-rays due to greater ionization density.^3^, ^4^, ^5^, ^6^ Collectively, this allows for the destruction of particularly radioresistant diseases, including hypoxic tumors. Studies suggest that concentrating high linear energy transfer (LET) particles into a hypoxic volume within a tumor or into a region containing tumor stem cells should lead to greater biological efficiency of PT and improvements in the therapeutic ratio.^7^, ^8^

Over many years, essential physical and radiobiological studies to enable patients to be treated with PT have been performed. Nowadays, in physics, current efforts focus on refining PT, emphasizing precision, efficiency, and cost-efficacy. Strategies include developing novel detectors, targeting moving tumors, adapting treatments to daily patient variations, reducing accelerator size, and exploring innovative approaches like gantry-less treatments, spatial irradiations (grid or minibeams), or UHDRs. For radiobiology, recent research underscores the point that this discipline can leverage the latest advancements in biotechnology to herald a new era of drug discovery and radiation therapy (RT) optimization in PT. Biological studies in PT must evolve from basic radiobiology to a deeper investigation of the mechanisms underlying its efficacy, aiming to expand the therapeutic window with new approaches in dose delivery regimens and evidence-based combined therapy approaches. This emerging advancement within radiobiology is poised to synergize with novel discoveries in oncology, such as immune system activation, radiosensitization/radioprotection, radiomics, biomarker discovery, genomically adjusted RT, and other innovative strategies.

A comprehensive exploration of the biological fundamentals of PT seeks to enhance comprehension and position PT as a frontrunner in oncology. This review delineates the areas of intense research in modern PT radiobiology, summarized in the Table, highlighting both advancements and knowledge gaps, covering methodologies, modeling, and clinical contexts to catalyze further exploration and discovery.

**Table:** Topics of current investigation in particle radiobiology.

Aspect	Concept
Beam delivery	
Ultrahigh dose rates	Short exposure times (∼ps-ms) result in higher normal tissue tolerance
Spatial fractionation	Higher doses can be better tolerated by normal tissues when delivered in a spatially fractionated manner (µm-mm exposure lengths)
Hypofractionation	Exploit good dose conformity to deliver ablative or immunogenic doses
New ions	High-LET ions (O, Ne) to target radioresistant (eg, hypoxic) volumes.
NTCP modeling and analysis	Understand the impact of dose distribution on normal tissue reactions (volume effects) for different endpoints, including second cancer induction
RBE in clinical endpoints	Optimize exploitation of RBE by modeling and translation between dose systems
Combined approaches	
Radioimmunotherapy	Radiation doses stimulate immune response, which can be converted into a strong antitumoral response by immune checkpoint inhibitors, eventually exhibiting *abscopal* response
Radiosensitizers	Enhance the DNA damage inflicted to DNA on the subcellular level, for example, by Auger electron emission of metallic nanoparticles, target specific DNA repair pathways to enhance tumor response without eliciting a normal tissue penalty
BNCT	Neutron irradiation of boron substances selectively targeted to tumor cells releases local alpha decay
Radiopharmaceuticals	Targeting residual tumor and metastases following the initial RT treatment of primary tumor
Accounting for individual radiation sensitivity	
Biomarkers and liquid biopsies	Monitor radiation action, for example, by considering DNA damage response markers; identify mutations in key genes that suggest conditional vulnerabilities to PT as opposed to conventional x-ray therapy
GARD	Consider an established genomic pattern to assess individual radiosensitivity

**Abbreviations: BNCT, boron neutron capture therapy; GARD, genomic-adjusted radiation dose; LET, linear energy transfer;** NTCP, Normal Tissue Complication Probability; **PT, particle therapy; RBE, relative biological effectiveness; and RT, radiation therapy.**

## Radiobiology of new irradiation modalities

### FLASH

Ultrahigh dose rate (UHDR, “FLASH”) RT is a breakthrough in cancer treatment with the potential to widen the therapeutic window.^9^ In preclinical models, the application of UHDRs (>40 Gy/s) of radiation has, in some cases, demonstrated a substantial reduction in normal tissue toxicity while maintaining tumor control. Recent advances in experimental capabilities have enabled FLASH studies with radiation types spanning from x-rays,^10^ electrons,^11^ clinical^12^ and low-energy protons,^13^ helium,^14^ and carbon ions.^15^, ^16^, ^17^, ^18^ The use of charged particles might enable the attainment of high dose rates more readily in clinical settings as compared to photons, and ongoing clinical trials are investigating the effectiveness of FLASH with protons and electrons.^19^, ^20^ While electrons offer a larger flexibility in terms of achievable dose rates, protons and heavier ions are seen as the more direct way toward clinical translation for deep-seated tumors.

FLASH experiments with heavier ions can now be performed inside the spread-out Bragg peak via the application of 3D range modulators, leading to studies assessing the maintenance of FLASH effects with high LET particles. Although the biophysical models attempting to explain the FLASH effect were predicting a loss of the sparing effect at high LET, recent experiments demonstrated persistent normal tissue-sparing effects in both in vitro and in vivo models.^14^, ^17^, ^18^ In addition, a study with ^12^C FLASH irradiations revealed a unique feature, notably the apparent suppression of distant metastases.^18^ These recent experiments with UHDR ^12^C beams have laid the groundwork for preclinical tests with ^16^O, or even ^20^Ne ions.

As the interest in FLASH radiobiology surged, it became clear that the existing understanding of tissue responses to radiation fails to explain the benefits of FLASH. Furthermore, although there is substantial evidence supporting the normal tissue-sparing effects of UHDR treatments, some irradiations with electrons and protons have demonstrated no such benefit.^21^, ^22^ The traditional understanding of dose rate effects, primarily based on chronic low-dose-rate exposures, was challenged by FLASH-RT's unique outcomes,^23^ suggesting a departure from reliance on DNA double-strand break (DSB) repair kinetics. Hypotheses such as increased free radical recombination/diffusion^24^ and oxygen depletion initially gained traction^25^ but were later countered by evidence showing insufficient oxygen reduction to confer biological benefits.^26^, ^27^ Alternative hypotheses, including protection of stem cell niches,^28^ dose-rate dependent changes in lipid peroxidation^29^ or other dose-rate-responsive molecules,^30^ and the impact on blood volume irradiation,^31^ which may be due to sparing some immune cells, have emerged but lack conclusive experimental validation.

Although the underlying mechanisms that drive the FLASH effect have not been fully explained, we believe the prospects of this novel technique as a common RT modality will be moved forward as the biology is elucidated and clinical trials are completed. To advance the understanding of the FLASH effect, additional experimental data are needed, both in vitro and in vivo, including data examining dose rate and other treatment parameters relevant to each radiation modality.

### Spatially fractionated radiation therapy

In spatially fractionated radiation therapy (SFRT), a heterogeneous pattern of radiation is delivered to tissues by creating regions with high (peaks) and low (valleys) doses, resulting in reduced normal tissue toxicities.^32^ The beam size is inversely correlated with the maximal doses tolerated by normal tissues.^33^, ^34^ In this context, minibeam radiation therapy (MBRT) utilizes planar beamlets with widths ranging from 0.3 to 1 mm. Compared to clinical SFRT techniques like grid and lattice RT (hot spots of 1-2 cm^2^), MBRT allows for smaller beam sizes that allow for increased doses to be delivered to a tumor.

The superior tissue-sparing capacities of proton minibeam radiation therapy (pMBRT) were demonstrated in preclinical studies with peak doses as high as 100 Gy, with no significant neurotoxicity in cranial irradiation on evaluation of memory impairment and histopathology,^35^, ^36^, ^37^ skin toxicity,^38^, ^39^ and, in thoracic irradiation, lung fibrosis.^40^ X-ray MBRT has shown a higher therapeutic index compared to that seen when an equivalent homogeneous dose is used against a rat glioblastoma model^41^, ^42^, ^43^ and de novo brain tumors in canine patients.^44^ The biological mechanisms underlying SFRT are under study. Activation of bystander cell-to-cell communication^45^ and an antitumor immune response seem key for tumor eradication with MBRT.^46^

Recently, pMBRT was proposed as a method to enable the delivery of MBRT to deep-seated tumors.^47^ Importantly, pMBRT has shown a similar tumor control capacity in high-grade glioblastoma orthotopic models in rats to highly toxic curative broad-beam doses.^37^, ^48^, ^49^ Integrating pMBRT with temporal fractionation in a crossed-beam approach appears to be the most effective approach to date.^50^

These promising in vivo results have led to the exploration of heavy-ion MBRT, including C, Ne, and Ar. While the latter ions are particularly efficient in treating hypoxic tumors, they also induce normal tissue toxicities,^51^ which may be mitigated by MBRT. Additionally, their physical scattering properties are ideal for maintaining the minibeam spatial dose pattern at greater depths in tissues than possible with protons.^52^ Neon MBRT has shown substantially lower skin tissue toxicities^53^ than Ne-broad beam RT, with significant tumor growth delay in a mouse sarcoma model despite 75% of the tumor receiving <2 Gy (forthcoming data). A similar sparing of skin toxicity was also shown using Li-7 ions.^54^ MBRT with high-LET charged particles offers significant promise for improving the therapeutic index in PT, especially for tumors near organs at risk. More preclinical data are necessary to unravel the mechanism underlying MBRT and to explore potential synergies with PT, including the hypothesis of enhanced immune activation. Clinical translation of MBRT in the modern era is in its early stages; the authors are aware of efforts in photon and proton modalities, although currently there are no published results.

### Boron neutron capture therapy

The principle of boron neutron capture therapy (BNCT) lies in the capture reaction of a thermal neutron by the boron ^10^B isotope, resulting in the production of a high-LET alpha particle (^4^He) and a recoiling lithium (^7^Li) nucleus. Due to the small tissue range of alpha particles (5-9 µm), the damage is primarily restricted to cancer cells where ^10^B atoms are preferentially delivered.^55^, ^56^ Clinical interest in BNCT spans a number of malignancies as high-grade gliomas,^57^, ^58^ cerebral metastases,^59^ primary cutaneous melanomas,^60^ and recently expanding also to liver and head and neck cancers.^61^, ^62^

Due to its complex radiation composition, BNCT's biological effects cannot be solely explained by absorbed dose. A better understanding of BNCT biological effects necessitates research using biological models and clinical trials integrating responses with known radiobiology. The conventional procedure for computing photon-equivalent doses in BNCT consists of adding the contributions of the individual radiation types to the total absorbed dose, each one weighed by a fixed factor, independent of dose and dose-rate.^63^ The currently used "weights" are RBE and compound biological effectiveness, obtained from reference cell survival experiments with γ-rays or x-rays. Patient treatment planning, despite the selected model, depends on reliable radiobiological data. It is thus crucial to report not only the photon-equivalent but also the total absorbed doses, including its components for the neutron dose used, the neutron source's spectrum, as well as the values of boron concentration in blood and tissues.

Boron neutron capture therapy efficacy strongly depends on the selective delivery and accumulation of ^10^B in tumors, achieved through boron carriers characterized by high tumor uptake and rapid clearance from blood and healthy tissues.^61^, ^64^ There are currently 3 constantly refined generations of boron compounds. The first generation includes boric acid and its derivatives; the second consists of boronophenylalanine and sodium mercaptan decahydro-closo-dodecaborate; and in the last decade, boron carrier development has taken principally 2 directions: small boron molecules and boron-conjugated biological complexes that represent the third generation.^62^

Studies pertaining to the use of fast neutron RT have contributed to a better understanding of the improved biological efficiency of particles in comparison to x-rays.^64^ With the emergence of accelerator-based BNCT, which will facilitate clinical trials in the traditional hospital setting, and improved drug delivery approaches under investigation, BNCT is primed to emerge as an important area of preclinical and clinical PT research in the years ahead.

### Radiopharmaceutical therapy

Radiopharmaceutical therapy (RPT) uses radionuclides emitting α-particle or β-particle, Auger electrons, and γ-rays,^65^ in combination with targeting vectors (including peptides, antibodies or their fragments, scaffolds, and small molecules) against a range of tissue-specific tumor biomarkers that maximize the localization of these radionuclides at the site of disease, while sparing normal tissues.^66^, ^67^

There are several critical differences between RPT and external beam PT.^68^ Radiopharmaceutical therapy dosimetry is less well-defined^69^ due to a heterogeneous dose distribution in tissues and organs. While in RT absorbed doses may be directly measured or calculated with knowledge of the particle field and patient anatomy, in RPT both radiation transport calculations and the pharmacokinetics of the administered radiopharmaceutical are required.^65^ The Medical Internal Radiation Dose Committee formalism for estimating absorbed doses in nuclear medicine imaging^66^ has been adapted for alpha-particle emitters in RPT.^67^ Standardization, advancements in imaging techniques, and rigorous dosimetry, along with clinical reporting, are crucial for assessing the RBE for different tissues and agents used in RPT and for facilitating its use alone or in combination with other modalities.^68^ Second, RPT typical dose rates (<0.5 Gy/h) are nearly 2 orders of magnitude lower than conventional PT, influencing the biological response to radiation and the potential for bystander effects.^70^

Moreover, the radiation penetration depth differs between radiopharmaceuticals and PT. Both α-particles and Auger electrons have a high LET and consequently deposit their energy over micrometer (28-100 µm) and nanometer (<500 nm) ranges, respectively, compared to the millimeter (0.5-10 mm) range associated with β-particles.^71^, ^72^ This impacts the microscale distribution of radiation dose within the target volume and surrounding tissues. Lastly, RPT involves continuous radiation exposure with an exponential decay over hours to days/weeks, whereas therapy with particles is typically delivered over a shorter period, which may result in distinct radiobiological effects.

Several studies have investigated the combination of external RT with RPT.^73^, ^74^, ^75^ This approach aims to deliver precise high doses to the primary tumor using external RT while also targeting residual tumor and micrometastases with systemic RPT. By combining these modalities, the protection of normal tissues surrounding tumors during external RT and dose-limiting organs such as kidneys and bone marrow during RPT can be better achieved. Novel therapeutic modalities like carbon ion RT (CIRT) are being explored in combination with RPT in tumor cells.^76^ Carbon ion RT has been shown to uniquely activate immune responses, DNA damage pathways, and cell-cycle control mechanisms, enhancing the effects of RPT agents.^75^

Despite early forays, the translation of combination of RPT and external R into clinical practice has not gained significant momentum. This may be attributed to the complexity of dose estimations, as well as legal and administrative challenges in certain countries, particularly in the context of radiation treatment. However, with recent advancements in RPT and improved dosimetry protocols, further exploration of combining RPT with external RT is warranted, offering the potential for improved treatment outcomes and enhanced patient care.

### Particle stereotactic irradiation

Stereotactic body radiation therapy (SBRT), also known as stereotactic ablative radiation therapy or radiosurgery when delivered in a single fraction, endeavors to deliver a wholly tumoricidal, ablative dose to a tumor target. The radiobiology associated with doses per fraction of >8 to 10 Gy differs significantly from conventional fractionation, with tumoricidal and normal tissue effects unexplained by the linear-quadratic model.^77^, ^78^ With conventional RT, it is generally accepted that most of the effects arise from unrepaired radiation-induced DNA damage, which results in mitotic death. In contrast, SBRT effects appear to be mediated by multifactorial indirect cell killing.^79^ First, vascular effects may drive excess tumor cell death as data have shown a differential effect on dysfunctional tumor vessels with endothelial apoptosis becoming significant above a ∼8 to 10 Gy single dose threshold (microvascular disruption resulting in death of the tissue supplied by that vasculature).^80^, ^81^ Second, large fractional doses with hypofractionation to some degree overcome hypoxia and radioresistance.^81^ Third, high fractional doses of radiation also seem to play a role in stimulating an immune response by releasing tumor antigens and inducing specific tumor responses.^82^ Finally, radiation-induced stem cell depletion is also likely important as stem cells can migrate into the radioablated tissue from neighboring undamaged tissue.^83^

Hypofractionation with Charged Particle Therapy (CPT) is promising.^84^ Based upon the seminal findings of Ando et al,^85^ who described a distinct advantage in treating a rat tumor with limited fraction numbers and high dose per fraction in the high LET region of a carbon ion beam compared to the limited adverse normal tissue response of the skin over the tumor site led to a number of clinical trials in different tumor types. For a review of those results, see.^84^, ^86^ An evaluating panel of Quantum Science and Technology clinical trials suggested that hypofractionation be accelerated because of the favorable outcomes in radioresistant tumors as well as providing greater access to the technology.^86^

Of concern, though, is the penumbra effects of smaller particles, such as proton RT, which may result in reduced low-dose delivery to surrounding healthy tissue but less sharp ablative dose fall-off outside the target tumor. Whether additional radiobiological mechanisms are involved is an area of investigation, particularly regarding LET deposition within the tumor target. Conventional conformal SBRT techniques may result in an LET distribution disproportionately deposited in tissues distal to the tumor along the beam path if LET-painting methods are not adequately applied.^8^ Limitations to wider implementation of particle stereotactic irradiation^87^ include uncertainties about dose computation, measurement, and radiobiological effects. Experience with protons is paradoxical: Once a classical/historical indication for pituitary tumors and vascular disorders (arteriovenuos malfromation) in the 1950s, the dosimetric limitations of small beams have slowed the implementation of modern particle SBRT for field sizes below 3 cm in the smallest axis. Detector design and algorithmic dose computational tools are currently an area of active investigation with promising proton stereotactic irradiation data.

### New ions

While most radiobiological studies in PT focus on proton or CIRT treatments, ongoing research aims to explore alternative radiation modalities, building on efforts initiated in the 1970s during trials at Lawrence Berkeley National Laboratory and followed by decades of advancements in both physics and biology. To this end, ions heavier than carbon, particularly ^16^O,^88^ have been considered appealing for targeting radioresistant and hypoxic tumors. Their increased oxygen enhancement ratio, associated with increased LET values in the target, makes them less sensitive to the presence of hypoxic regions within the tumor. However, the use of these ions remains hampered by the increased risk of normal tissue toxicities associated with their high RBE.

Combinations with lighter ion beams, such as protons or ^4^He, where different ions, based on their physical and biological properties, are directed to certain parts of the tumors, may mitigate these risks.^89^ However, the efficacy of such approaches remains uncertain due to a lack of clinical and preclinical data, partly attributable to the absence of animal models suitable for testing field redistributions. Additionally, high-LET particle oxygen enhancement ratio (OER) models heavily rely on in vitro data, introducing uncertainties to in vivo effect estimation. Further experimental efforts expanding to preclinical models are thus crucial to refine OER modeling and understand the biological, chemical, and physical aspects of hypoxia radioresistance.

Heavy-ion facilities are cost-prohibitive in comparison with conventional or proton therapy, hampering their clinical adoption. Light ions, such as ^4^He or ^7^Li, may represent a good compromise between proton and CIRT in terms of cost-effectiveness. Furthermore, while ^4^He beams have already been recently integrated into clinical practice,^90^, ^91^ Monte Carlo models suggest that their radioactive isotopes can offer higher Bragg peak doses without an increase of the dose in the plateau region,^92^ though the clinical translation of helium monotherapy has not been verified to date. Although their production at sufficient intensities remains challenging in clinical settings, modern high-intensity accelerator facilities already offer the possibility of first pilot studies.

## Multimodal radiation therapy

Numerous novelties within systemic therapy and photon RT may provide robust translation opportunities for combinatorial effect with PT, above and beyond what is possible with conventional RT.

### Charged particle therapy in combination with immunotherapies

Immunotherapies, mainly immune checkpoint inhibitors (ICI), are currently a component of standard of care in several cancer types and are often included in cancer therapy regimens in combination with other systemic and local therapies, including RT. The combination of immune and RT has shown promising results in patients who did not respond to other therapies.^93^, ^94^ The mechanistic basis for such a combination is RT immunogenicity, that is, the creation of de novo antigens (referred to as antigenicity) and the release of factors attracting and activating immune cells (ie, adjuvanticity).^95^ The induced immunogenicity is then boosted with (neo)adjuvant immunotherapies, mostly ICI.

However, only a fraction of patients responds to such combinations, and recent studies combining photon RT with ICI have failed to meet their primary endpoints.^96^ The design of these studies has been criticized, showing the importance of clinical trial design for combined treatment with immunotherapies, and leaving significant room for improvement. From a biological perspective, the lack of signal from these trials could be influenced by the decision to irradiate lymph nodes. While they are intentionally irradiated during therapy to eradicate putative metastases, recent experimental data have shown that sparing lymph nodes is pivotal for an efficient immune response following RT.^97^ From this perspective, PT may provide beneficial properties for combining radiation with immunotherapy due to the physical and biological features of particles.^98^, ^99^ The high precision of beam delivery allows for improved sparing of circulating lymphocytes (and other lymphoid organs-at-risk like bone marrow, thymus, or spleen), and hence immune cells are available for an immune response. This is supported by evidence showing a lower degree of lymphopenia following PT.^99^ The level of immunogenicity of the cellular response to RT is crucial and mainly depends on 2 factors, that is, antigenicity (neoantigen repertoire triggered by radiation exposure) and adjuvanticity (release of immunogenic danger signals during cell death or stress response).^100^ In this light, charged particles, especially CIRT, are discussed to be of advantage due to an increased RBE, different cell death patterns, and clustered and more complex DNA damage. Ultimately, the question is whether immune “cold” tumors can better be turned into “hot” tumors by PT as compared to conventional RT.^101^, ^102^ While generally the mechanisms of RT on immunogenicity are not well understood (eg, with respect to dose or fractionation schemes), this is particularly true for PT. Other open questions are related to the sequence of administration of immunotherapies relative to RT.^103^

Two interesting new approaches in RT regimens propose to bootstrap immune-related features of tumors via the host or the treatment, by changing patient fractionation schemes either temporally or spatially. In preclinical animal models, the personalized ultrafractionated stereotactic adaptive radiation therapy (PULSAR) regimen separated fractions by several days, thereby allowing time for adaptation within tumor tissue as well as with respect to the immune response.^104^ In human patients, this gap between fractions can be weeks. This follows the hypothesis that repeated longitudinal exposure to tumor antigens may amplify the adaptive immune response and thereby improve immune control of metastatic cancer disease.^105^ Personalized ultrafractionated stereotactic adaptive radiation therapy has not been used with PT yet, but the ultrafractionation and a hypothesized improved immune response render PT attractive for implementation in PULSAR.

One spatial fractionation approach is called stereotactic body RT-based partial tumor irradiation targeting hypoxic segments of bulky tumors (SBRT-PATHY), demonstrating promising initial outcomes. The authors are investigating apparently improved exploitation of bystander and abscopal effects, aiming to specifically target the hypoxic and immunosuppressive parts in the tumor microenvironment while sparing the peritumoral immunological microenvironment, including nearby tissues, blood-lymphatic vessels, and lymph nodes.^106^, ^107^ This is hypothesized to enhance immune response. Based upon these promising results, carbon-PATHY takes advantage of the proposed mechanisms above along with the advantages of carbon ions, including a reduced OER. Indeed, the approach is being tested with CIRT in clinics with promising results.^108^, ^109^

In summary, the combination of charged particles and immunotherapy may be powerful but many questions remain to be answered. It is pivotal that particle radiobiologists work closely together with immunologists to shed light on the immune-related mechanisms of PT. Initial forays into clinical translation have begun, but poor access to PT centers limits robust study of these observed effects.

### Radiosensitizers

Radiosensitizers are compounds that augment the potency of ionizing radiation in eradicating tumor cells, typically measured by their enhancement ratio.^110^, ^111^, ^112^ The interest in applying radiosensitizers in combination with particles is growing due to the hypothetical ability to reduce total patient dose while increasing tumor control probability.^113^ Considering the basic biological mechanisms of action, radiosensitizers can be classified into 5 categories: (1) suppression of intracellular thiols or other endogenous radioprotective substances, (2) formation of cytotoxic substances by radiolysis of the radiosensitizer, (3) inhibitors of DNA repair, (4) thymine analogs that can incorporate into DNA, and (5) oxygen mimetics that have electrophilic activity.^114^, ^115^ Regarding their different structures, radiosensitizers can be classified as small molecules, macromolecules, or nanomaterials.^116^, ^117^ Some small molecules that are currently being investigated include peptides, miRNAs, siRNAs, and oligonucleotides.^114^, ^117^ One strategy for radiosensitizers has been to repurpose drugs with approved noncancer indications and review their efficacy in combination with radiation or chemoradiation through clinical trials, allowing rapid trial evaluation without the need for early-phase, regulatory-approval studies.^118^ Other radiosensitizers, such as naturally occurring products (phytocompounds), are also undergoing clinical trials.^119^ Examples include curcumin, resveratrol, dihydroartemisinin, and paclitaxel.^114^, ^117^

Furthermore, the use of nanoparticles (NPs) has drawn attention in recent years.^120^, ^121^, ^122^ Technological advances in NP synthesis/functionalization have led to significant advances in molecular detection, imaging, targeting, multifunctional therapeutics, and prevention and control of diseases.^121^ In particular, the application of metallic NP has received growing attention as a radiosensitizer in PT.^113^, ^114^, ^120^ Some NPs with proven radiosensitizing effects are made of noble metals (eg, gold, silver, and platinum) or heavy metals (eg, gadolinium, hafnium, tantalum, tungsten, and bismuth).^114^, ^122^ The radiosensitization potential depends on numerous factors: cell line, NP type and size, concentration, coating, intracellular localization, and energy and nature of radiation.^122^ Different methodologies have been proposed to study the radiobiological effect of these materials.^123^ Noble metal nanomaterials have been extensively studied using x-rays.^113^, ^114^, ^124^ However, the mechanistic explanation for the local dose enhancement provided for photons is different from that for ions, where the dose is already highly localized along the tracks, and an extremely high local dose is required to increase the damage further, without even accounting for overkill effects. In this case, the enhancement of the radiation effects is not yet fully understood.^113^ Similar mechanisms have been reported, such as an increase in secondary electrons together with the increase in reactive oxygen species formation, oxidative stress, inhibition of DNA repair, changes in the cell cycle and organelle function that increase cytotoxicity, inhibition of the expression of radiation resistance genes, or the promotion of expression of radiation-sensitive genes.^114^, ^124^ Considering that NPs induce oxidative stress and inflammation, an evaluation of ion release and subsequent biological responses, oxidative stress, and inflammation is important for nanotoxicity.^125^ Also, the selective delivery of NPs could be passive or via delivery systems with tumor-specific agents.^113^, ^126^ Moreover, antisense oligonucleotide genetically loaded NPs can also be designed for use via gene radiosensitization.^126^

Despite the advantages of nanomaterials, few have been translated into clinical trials.^127^, ^128^ To confirm the uptake in correct locations, NPs that have translated to clinical trials have tended to be “theranostic“ agents, that is, visible on diagnostic images prior to irradiation.^129^, ^130^, ^131^, ^132^, ^133^ Although there has been great interest in the use of noble metal NPs, investigation is still needed to control and optimize their effect before translation into clinical trials. Furthermore, other NPs were found to have radioprotector effects instead,^134^, ^135^, ^136^, ^137^ which is a potentially alternative approach. In addition, while the oxygenation of the tissues may play a significant role, the OER effect associated with the presence of NP has not been considered.^113^ Despite that, some compounds that mimic oxygen capabilities are being investigated.^114^ Moreover, hypoxia-specific cytotoxins could be used for overcoming the radioresistance of hypoxia tumors.^114^

### Prediction and prognosis in radiation oncology

In medicine, prediction can be directed at 3 aspects of an individual’s health status. One can predict the risk for a given cancer, one can predict the response to a given therapy and one can predict the risk for disease recurrence. Prognosis, on the other hand, speaks only to the overall outcome regardless of therapy or perhaps to standard therapy and does not rely on the data one might use for prediction. Prediction relies on biomarkers, a term often used casually and which is defined by the FDA as a validated characteristic that is objectively measured as indicators of health, disease, or a response to an exposure or intervention, including therapeutic interventions.

Precision medicine in radiation oncology can be broadly divided into efforts by the physical sciences, that is, not only the application of imaging-based physical mapping and precise localization of target tissue but also the heterogeneous and unique features derived from image analysis, so-called radiomics. Biological approaches that quantify the entire collection of specific categories of biological molecules that can translate into the dynamic function of a cell, tissue, or organism are called radiogenomics.

Omics can be broadly classified at the whole genome level, where single nucleotide polymorphisms (SNPs) are examined, and by copy number variation, where chromosome structure is examined to identify gene copy number, inversions, deletions, and other genomic rearrangements. Just below the genome level is transcriptomics—the characterization of all transcribed RNA species whether translated into protein or not; proteomics—the characterization of proteins; epigenomics—the evaluation of the locations and functions of chemical tags, DNA methylation as an example, along the genome; and metabolomics, where the metabolites generated by cellular function are characterized. In radiation oncology, most effort in omics analysis has focused on tumor transcriptomics; however, unlike other disciplines in medicine, radiation oncology has also examined the responses of normal tissue given that treatment outcomes are not only based upon tumor control probability but also the probability of normal tissue complications. Furthermore, assay development besides following more traditional approaches to omics analysis focused on direct analysis of tumor or normal tissues, new less invasive approaches that examine circulating factors such as circulating fragmented tumor DNA (ctDNA), circulating miRNA, or circulating exosomal cargo (DNA, RNA, and proteins) are gaining favor based upon increased computational power for next generation sequencing and the limited invasiveness of fluid (blood, urine, sputum)-based assays.

#### Indicators of individual radiosensitivity

Individual radiation sensitivity (iRS) characterizes the specific tissue/cellular response to ionizing radiation and has a significant influence on the variables affecting late RT toxicity. Like other biological processes, iRS is represented as a Gaussian curve, with patients with very severe tissue responses but with a broadly normal phenotype at the left of this curve.^138^ At the molecular level, damage to DNA and biomolecules, DNA repair pathways, cell death, as well as oxidative stress strongly impact the RT toxicities and the sensitivity to specific DNA damaging systemic therapies. As a possible biomarker for radiosensitivity, the relationship between genes, their products, or regulators has been largely investigated.^139^ However, implementing these potential biomarkers in the RT workflow remains challenging. Several approaches have been developed to identify biomarkers for patients undergoing RT, including preclinical and clinical studies, agnostic approaches like high-throughput proteomics or genome wide CRISPR screenings or genomic analysis of resistant cellular models.^140^, ^141^, ^142^

Most known biomarkers are related to the Hallmarks of Radiobiology: DNA damage repair, tumor cell redistribution in the cell cycle, repopulation, reoxygenation, and radiosensitivity. For instance, the functional analysis of subnuclear DNA damage response (DDR) foci in tumor tissues, peripheral blood lymphocytes,^143^ or circulating tumor cells (CTCs) might predict iRS. These foci are dynamic multiprotein complexes centered around a DSB. They appear as "dots" in cells or tissues under immunofluorescence and have a structure related to the entity of DNA damage, the cell cycle stage, and local chromatin structure. These sites represent local spreading and intensification of signal after DNA damage and function as a "toolbox" to support downstream function of the DDR including DNA repair and cell cycle checkpoint responses.^144^, ^145^ The primary advantage of these DDR foci as an iRS functional biomarker is their ability to disclose the presence of repair deficiencies, including defects from changes in signal transduction pathways,^146^ epigenetic events, or gene mutations.^138^, ^147^, ^148^, ^149^ These foci offer a comprehensive evaluation of DDR network performance without requiring knowledge of every network component—many of which are currently unknown.^145^ It is possible to imagine creating mechanism-based "DDR foci signatures" where network nodes are represented by the presence of key proteins such as γ-H2AX, 53BP1, BRCA1/2, RAD51, FANCD2, or others. Indeed, there is a linear correlation between cell survival after irradiation and residual γ-H2AX, making them a surrogate of radiosensitivity,^150^, ^151^, ^152^ as well as tumor bioptic predictive biomarkers of radiosensitization caused by a molecular targeted agent.^153^, ^154^ Compared to γ-H2AX, 53BP1 seems to be more accurate in identifying DSBs making it an alternative biomarker.^155^, ^156^ Other mentioned proteins are employed as a surrogate of homologous recombination repair and nonhomologous end joining activity and might contribute to the additive toxicity in the combination approaches, also after CIRT.^2^, ^157^

It is also noteworthy that cell lines with homologous recombination repair mutations have decreased OERs compared to normoxic scenarios, with irradiation in hypoxia leading to the creation of more DNA interstrand cross-link formation. Effects of HR on DNA-protein cross-links are still a possibility, as there is strong evidence of higher yields in hypoxic environments. Because of hypoxic dependency and proliferation, targeting HR to promote radiosensitivity might provide for partial tumor selectivity.^158^ Moreover, hypoxia-induced changes in DNA mismatch and base excision repair lead to chromosomal instability, providing the basis for a “mutator” phenotype.^158^ The variation in hypoxia might be traceable in gene expression signatures (ie, HIF-1)^159^, ^160^ and in the meantime through the analysis of radiomics and radiogenomic features,^161^, ^162^ serving as promising noninvasive/minimal-invasive predictive biomarkers of radiosensitivity/resistance.

#### Fluid-based biomarker development

Single nucleotide polymorphisms in DDR genes identified in peripheral blood cells have been shown to be associated with iRS, with a tissue specificity for each genetic determinant and “linkage disequilibrium” for which some SNPs can catch most of a regional genetic variation.^138^ One of the most analyzed is the missense ATM SNP rs1801516 that has shown to correlate with high risk of post-RT fibrosis, especially in breast and prostate cancers.^163^, ^164^ Moreover, for some SNPs,^165^, ^166^ only the heterozygous state confers their respective radioprotective and sensitizing effects. The combination of polymorphisms in different alleles seems to be a feasible approach to assess the iRS.^167^ Although encouraging, the SNPs model was rarely applied to the validation cohorts, and it is difficult to obtain significant statistical power to assess isolated SNPs. Through the European REQUITE project, most advancements have been made in identifying SNPs linked to late toxicities in breast and prostate cancer.^168^ The combination of SNPs with the dosimetric parameters (Normal Tissue Complication Probability (NTCP) and LET distributions), clinical risk factors, and comorbidities linked to high intrinsic radiosensitivity (ie, radiosensitive syndromes) might be helpful in defining patient-tailored iRS.

Besides following more traditional approaches to omics analysis focused on direct analysis of tumor or normal tissues, new less invasive approaches that examine circulating factors such as ctDNA, circulating miRNA, or circulating exosomal cargo (DNA, RNA, and proteins) are gaining favor based upon increased computational power for next generation sequencing necessary to analyze ctDNA from circulating free DNA and the limited invasiveness of fluid (blood, urine, sputum)-based assays.

The assessment of ctDNA found in blood and urine can also take a different strategy from the approaches described above in that the analysis is designed to detect cancers earlier^169^, ^170^ and predict therapeutic response but also to detect minimal residual disease (MRD).^171^, ^172^, ^173^ By tracking specific biomarkers, the overall response to therapy and, subsequently, MRD can be followed, and, most importantly, there is the potential to identify disease recurrence well before pathophysiologic indicators of recurrence. Exosomal cargo (DNA, RNA protein, miRNA)^174^, ^175^, ^176^ and circulating miRNAs^177^, ^178^ are also advancing in the search for fluid-based biomarkers of radiotherapeutic response and MRD.

#### Circulating tumor cells

Isolating CTCs from patients' blood has been a challenge for researchers, which has led to only a few studies to detect the effects of radiation on the CTCs. However, being able to isolate and culture these cells in vitro could provide enormous benefits and is worth exploring. Monitoring CTCs in cancer patients undergoing RT could provide new insights into how metastatic spread is influenced by radiation and vice versa. Additionally, CTCs could become a valuable source of biomarkers, a liquid biopsy, used to study treatment response. A relapse during RT has been associated with an increase in the number of CTCs and thus, their count could be used as predictive biomarker for clinical trials.^179^

Conventional radiation has been shown to disrupt the primary tumor vasculature, potentially increasing the dissemination of CTCs. For instance, during early-phase RT an increase in CTC number in non–small cell lung cancer (NSCLC) was observed.^180^ Photon radiation can also induce epithelial-mesenchymal transition, which could potentially lead to dormant CTCs awakening, fostering proliferation, resistance, and metastasis. The shedding of mesenchymal marker-expressing CTCs was observed during NSCLC RT, potentially informing new strategies to monitor metastatic spread post treatment.^180^, ^181^ In a study of a large cohort of patients with early-stage breast cancer, it was reported that CTC-positive patients that received RT had a longer survival rate compared with nontreated patients.^181^ Radiation therapy in combination with chemotherapy has been shown to reduce the number of head and neck squamous cell carcinoma and prostate CTCs.^182^, ^183^, ^184^ The number of CTCs, indeed, is a suitable marker for the response to x-rays RT,^185^, ^186^ but there is a lack of publications that examine in detail the influence of RT on CTC genotype. Furthermore, it should be noted that almost all previous studies on RT and CTCs were focused on x-rays with some exceptions dealing with protons and carbon ions. Exploring the quantity and nurturing of CTCs in vitro postparticle irradiation could unveil novel markers for probing therapy development. This exploration may also illuminate diverse mechanisms underlying metastasis formation in both conventional and particle RT.

Besides CTCs or other genomic content in blood, blood chemistry is also of increased interest for stratifying patients and predicting treatment response.^187^ In this scenario, the blood cell count is an easily and often available parameter that might reflect the inflammatory response^188^, ^189^ as well as the oxygenation status.^190^ Recent literature showed their prognostic role in several tumors and in different RT settings, including high LET.^191^ Routinely available in this context is also the blood glycemic status that, when increased, seems related to more aggressive tumor phenotype.^192^, ^193^

#### Genomic-adjusted radiation dose

The concept of genomic-adjusted radiation dose (GARD)^194^ proposes to exploit information on genomic expression obtained from a tumor biopsy or a liquid biopsy, like the CTCs extracted from a patients' blood, to estimate the radioresistance of the malignancy affecting a specific patient. In this framework, it is then theoretically possible to set up a personalized dose prescription for each single patient for whom the genomic expression test is available. This would comply with the ideal need to deliver a treatment that is as far as possible tailored to tumor biology, thus aiming at higher effectiveness. An approach of this type could be pan-cancer, that is, tumor site-agnostic or tumor site-specific, where the genomic panel used to interrogate the tumor is unique to the tumor site.

A pan-cancer approach was developed by ultimately combining the surviving fraction at 2 Gy for 48 cancer cell lines from the NCI-60 panel with a biomarker discovery platform that, starting from an initial set of 500 genes, identified 10 hub genes whose expression could be used to estimate surviving fraction at 2 Gy.^195^ This Radiosensitivity Index (RSI) appeared to be strongly related to radiosensitivity. Subsequently, the RSI algorithm was then tested using 3 gene expression data sets of patients treated with chemoradiation to predict tumor response and prognosis,^196^ followed by examination of other tumor sites treated with, or not, radiation including breast,^197^ endometrial,^198^ and pancreatic cancers.^199^

To develop the concept of a genomic-adjusted radiation dose, the RSI was combined with the linear-quadratic model for cell survival, where the RSI value contributes to the *α* term, which includes the patient-specific treatment response. The GARD determined for a given patient is calculated as *nd(α + βd)*, with *n* number of fractions and *d* dose per fraction. The GARD was tested on several cohorts of patients previously receiving RT.^194^, ^200^ It was shown that, when accounting for the RSI, the variability in the GARD values determined was much larger than that due to the physical dose received. Namely, where a dose stratification was performed, there could be patients originally assigned to the low-dose group, who due to their intrinsic radiosensitivity might be better served in a higher dose group based upon their calculated GARD value, and vice versa. This was also corroborated by a later study, where GARD was found to be associated significantly with both time to first recurrence and overall survival.^200^

The proposal of GARD elicited positive feedback as well as criticism such as the patient populations used for analysis and criteria for defining recurrence, among others. The application of GARD in an era of increasing use of stereotactic ablative radiation therapy was also noted which would likely apply to hypofractionated CPT. Furthermore, a recent study based on a reanalysis of the original publication as well as on additional data raised skepticism on the possibility to use RSI as a robust tool to adjust dose prescriptions in RT,^201^ which was defended through correspondence with the original GARD proponents. Obviously, the limited data currently available do not allow drawing final conclusions of the clinical potential of the RSI and, therefore, of GARD as an effective tool for RT individualization. Importantly, there is at least 1 clinical trial (ie, NCT05528133 focused on triple-negative breast cancer) currently recruiting patients. The outcomes of this and similar studies will shed light on this promising as well as debated topic.

The RSI tool and the GARD approach were developed in the framework of photon RT using conventional fractionation. It is possible that gene expression is differentially modulated by different types of radiation such that extending GARD to CPT would require additional investigation. At the same time, it could be hypothesized that the RSI is associated with smaller interindividual variations in RBE. Nevertheless, it could be intriguing to investigate the possibility of combining the high spatial selectivity of particle beams with both their higher RBE and a genomic-based personalized dose prescription.

Besides GARD other biomarkers within a specific disease site have been developed and validated. This includes the radiation sensitivity signature for breast cancer.^202^ This 51 gene signature was independent of disease subclassification and outperformed all other clinicopathologic predictors of treatment response. The radiation sensitivity signature signature is strongly linked to cell cycle and DNA repair pathways. Similarly, the RadR signature, developed in HPV-head and neck squamous cell carcinoma was unique for the use of tumor and paired normal mucosal samples, cell lines, and genes associated with disease-free status after surgery and radiation.^203^ The resulting 13 gene signature was used in an integrated analysis along with genomic alterations, protein expression, and drug sensitivity. While the RadR score was associated with molecular classification, the median RadR score was capable of segregating patients treated by surgery plus radiochemotherapy based upon recurrence-free survival, but not those treated by surgery and chemotherapy, thus validating the specificity of RadR for radiation response. Neither of these signatures has been applied to PT, although there is potential to determine if these signatures are pan-RT, that is, apply to CPT as well as to x-rays.

Confounding the development of radiation response biomarkers via omics analysis are dosimetric and volumetric considerations that are rarely, if at all, accounted for. Hypofractionated schedules, like those often used with ^12^C or stereotactic or ultrafractionated schedules, may negatively impact the accuracy of some algorithms that were developed from information gleaned from patient populations treated with conventional 2 Gy/fraction exposures. There may be archived tissues from CPT trials with dosimetric and volumetric information along with clinical and pathologic information, where putative biomarkers could be tested for application in CPT, be it normal tissue or tumor response. Not addressed at all is the contention that age (cancer patients are generally older) and sex may bias biomarker screening.^204^

#### Radiomics

Genomic-adjusted radiation dose and RSI represent a possibility for biologically motivated patient stratification; however, they are associated with additional time, effort, and costs for the requisite biological analyses. Radiomics endeavors to utilize the diagnostic images gathered as part of routine clinical practice, such as computer tomography, magnetic resonance imaging, positron emission tomography, x-ray, and ultrasound, extract objective, quantitative image features from the image, and then analyze these features for correlation or prediction of clinical features, generally using machine learning.^205^, ^206^ As early as 2014, a clustering between certain image features and the tumor stage and histology was predicted for NSCLC tumors,^206^ paving the road for radiomics as potential biomarkers for diagnosis, prognosis, and prediction. To establish such a computerized model, 2 data sets are required: a training data set and a data set for validation. The more heterogeneous the data sets are (different clinics, countries, devices, etc), the more robust the model becomes, but consequently, more data sets (ie, patient images/data) are needed for model development. Particle Therapy Cooperative Group may be one such platform for the development of such databases of particle-treated patients.

Dosiomics extends radiomics by extracting useful features from 3-dimensional RT dose distributions for the prediction of treatment outcomes^207^, ^208^ or normal tissue responses.^209^ Indeed, dosiomics applied to skull base chordomas treated with CIRT revealed the association of these features with adverse outcomes.^210^ In addition, the dose-averaged LET in CIRT has been found to correlate with local recurrence in chondrosarcomas^211^ and sacral chordomas,^212^ leading to an increased interest in a combined RBE-based and LET-based treatment optimization. In this regard, a retrospective analysis^213^ using dosimetric-identified features to identify possible quantitative prognostic factors to predict local control in sacral chordomas suggested that features extracted from LET_d_ maps can be employed for patient stratification into high-risk or low-risk groups for disease recurrence.

Of particular interest for RT are delta-radiomics, where a longitudinal image sequence is analyzed for signature change. Longitudinal image sequences are continuously generated in RT through imaging diagnostics, during treatment, and as a control in the follow-up. Without additional burden for the patient, a prognosis for the treatment response could already be made during treatment and, if necessary, the treatment could be adjusted. To date, radiomic analyses have not been robustly linkable to biomedical processes, and efforts are underway to develop quality standards for model reporting, such as Transparent Reporting of a multivariable prediction model for Individual Prognosis or Diagnosis^214^ and the Radiomics Quality Score—RQS 2.0.^215^ Nevertheless, there is great potential for translational biomedical studies that attribute changes in image signatures to biological processes.

Recent research approaches have adapted classical imaging formats such as histological staining of tumor tissue by hematoxylin & eosin staining, used in routine diagnostics with radiomics.^162^ These histo-radiomics studies are predestined for preclinical trials with orthotopic and heterotopic tumor models or replacement methods such as the chorioallantoic membrane assay, which also allows xenotransplantation of primary and established cell and tissue cultures that grow into well-vascularized tumors for tumor microenvironment studies.^216^ The tumors can be sampled at different times after irradiation and examined classically by histology or with spatial omics such as Matrix-Assisted Laser Desorption Ionization imaging^217^ providing insights into biological processes. Combined radiochemotherapy is the standard of care for most tumors, meaning that not only RT alone but also combined approaches with established chemotherapies and new substances such as targeted drugs should be investigated. Here too, preclinical tumor models have an advantage over the clinic in terms of sample throughput and substance spectrum. Overall, (histo)-radiomics has great long-term potential to stratify patients and to make recommendations for dose, fractionation, radiation quality, and systemic therapies.

## Radiobiology model systems

Biological studies often rely on adherent cell monolayers or 2D cell cultures for their convenience and ease of maintenance. Such models have advanced the understanding of basic of cellular mechanisms post RT, including DNA damage and repair, survival, and cell death.^218^, ^219^ They also serve as effective tools for drug screening, particularly for evaluating radiosensitizing agents.^114^ Alternatively, ex vivo tissue slice cultures or patient-derived explants can be relatively easily prepared in replicates to study the effects of radiation in the same sample in an organotypic environment with preserved original tissue architecture. While long-term (several months) culture of rodent tissue slices has been reported, human glioblastoma slices could only be maintained for several days or weeks in some cases^220^, ^221^ with the quality of patient material being the limiting factor.

Other conventional models that have advanced cancer radiobiology research include animals and small animal RT research platforms.^222^, ^223^ Mouse models, for example, allow for mimicking systemic radiation effects in order to observe and evaluate the impact of acute and late normal tissue effects at the organismal level. Studies in mice have for instance shown that radiation does not only affects synaptic function and behavior^224^ but also causes a decrease in dendritic complexity, reduction in dendritic spines and synaptic plasticity in the brain, leading to cognitive dysfunction.^225^ Long-term radiation studies in rodents also showed increased vascular perforation leading to radiation myelopathy.^226^, ^227^ Unfortunately, animal findings may not fully translate to human clinical settings due to differences in genetics, morphology, anatomy, and metabolism,^228^ all of which are factors in the response to therapeutic treatments. In addition, animal studies also bring ethical considerations.

Recent advances in stem cell research and 3D cell/tissue technologies may circumvent these issues. Unlike spheroids, generated from immortalized cell lines or primary cells, organoids are derived from stem cells or primary tissue (ie, patient-derived organoids [PDOs]).^229^, ^230^, ^231^ The use of PDOs, which truly resemble the original tissue or tumor with its specific set of mutations and/or inflammatory characteristics, allows for predicting the efficacy of RT and optimizing personalized treatment strategies.^232^ While PDOs represent a patient-specific disease state and are generated in a short time, organoids are derived from induced pluripotent or embryonic stem cells. This circumvents the problem of limited availability of human samples and allows the generation of multiple organ-specific cell types and subtypes from the same genetic background as well as easier gene editing. Their organ-like properties bridge the gap between cell culture studies and clinical approaches. A recent study using human brain organoids showed that postradiation image changes, that is, contrast-enhancing lesions, can be attributed to the formation of aberrant blood-cerebrospinal fluid barrier or choroid plexus in response to altered NOTCH and WNT signaling involved in cell differentiation.^233^ Furthermore, human organoids can be maintained in long-term culture as organoid slices cultured at the air-liquid interface,^234^ allowing for studies of long-term radiation effects.

To support the biological application, engineers, together with biologists, are developing an organ-on-a-chip, microfluidics-based approach, which may open up even more possibilities for Tumor MicroEnvironment modeling^235^, ^236^, ^237^ and other applications within radiobiological research.^238^, ^239^, ^240^

While these model systems, summarized in the Figure, offer significant advantages in terms of controlled experimental conditions and the ability to mimic specific aspects of the tumor microenvironment, they may not fully replicate the intricate interactions and complexities found within living organisms.^241^ Furthermore, the dose of radiation applied can have different outcomes depending on the dynamic structure and function of the organs. However, despite these limitations, continued advancements in technology and methodology hold great promise for enhancing our understanding of radiation-induced effects and developing novel treatment strategies for conventional x-ray therapy as well as CPT.

**Figure:**
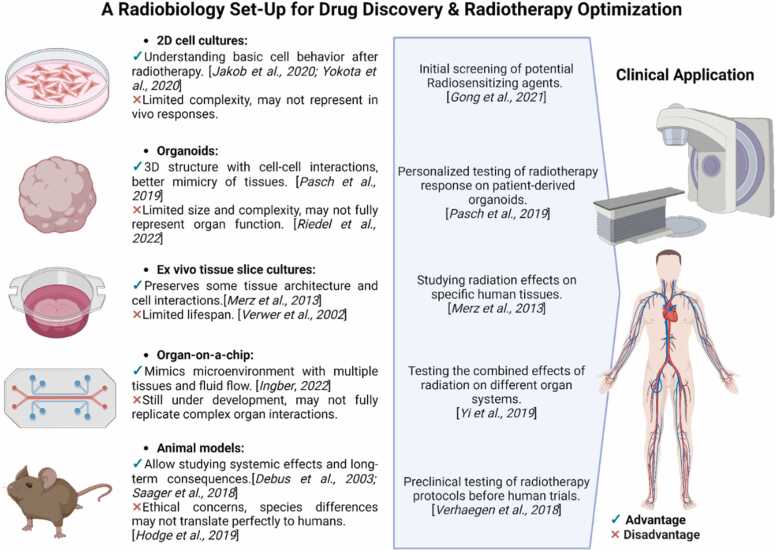
Different biological models according to their level of complexity, from cellular to tissue/organ to organism level, with their advantages and disadvantages and the possibilities they offer in drug discovery and radiation therapy optimization. Created with BioRender.com.

## Modeling

Mathematical models in radiation biology allow us to challenge underpinning mechanistic hypotheses which, once validated, can be used as tools to assess radiation effectiveness for treatment planning or other endpoints related to CPT. Here, we summarize selected fields of current interest in effects modeling for protons and heavier ions.

### Relative biological effectiveness modeling for treatment planning

The RBE depends on both physical (LET, energy, dose, and fraction number) and biological factors (α/β ratio, ratio of α’s), requiring a consistent description of these dependencies for complex radiation fields in a therapeutic setting. There are 2 opposing strategies to model RBE: (1) mechanistic models predicting RBE values by simulating selected underlying physical, chemical, and biological processes, while (2) empirical models fit analytical functions to experimental results and extrapolate them to new scenarios. In fact, most models exploit elements of both strategies.

A feature of ion radiation is that RBE increases with LET depending on ion type and α/β ratio before this trend is inverted due to overkill.^242^ Two mechanistic models are used in treatment planning: the local effect model (LEM I)^243^ and the modified microdosimetric kinetic model in Japan.^244^, ^245^ Model variants have been suggested (eg, LEM IV,^246^, ^247^ Mayo Clinic Florida-Microdosimetric Kinetic Model (MKM)^247^). An assessment of the MKM and other models^248^ inspired the creation of GSM2.^249^ Still other improvements are under discussion. Further mechanistic models also exist, for example, in nanodosimetry^250^^,^^251^ and machine learning-driven modeling.^252^, ^253^, ^254^ The advent of new heavy ion centers underlines a need for comparison of treatment plans optimized with different RBE models.^255^, ^256^ Issues in dose reporting have been addressed,^257^ but given the scope of possible future model diversification, discussing model-induced uncertainty in carbon ion therapy is of high relevance. The advent of new heavy ion centers underlines a need for comparison of treatment plans optimized with different RBE models.^255^, ^256^ Issues in dose reporting were addressed,^257^ but in the scope of possible future model diversification, discussing model-induced uncertainty in CIRT is critical.

In proton therapy, an RBE of 1.1 is usually employed. However, there is strong evidence from cell experiments and recent clinical indications for an increase in RBE with LET.^258^ A family of empirical models attempts to describe the LQ parameter dependence on LET and α/β.^259^, ^260^ The models differ in the expressions for the fitting parameters and the data used to obtain them, reflecting uncertainties in RBE determination. Additionally, there have been attempts to apply the LEM and MKM to proton therapy.^261^, ^262^, ^263^

Several pools of experimental RBE collections are becoming available, for example, the particle irradiation data ensemble^264^, ^265^, ^266^ and large single sets of experiments,^267^, ^268^ reflecting the richness of RBE systematics. These data sets are fundamental for tests of the models, which should be fitted to specific data and tested independently on other portions of the database (eg, different ions or LET regimes). Dedicated experiments can also be performed to confirm the model predictions, in particular when applying them to multiple situations (eg, varying dose rates and different endpoints).

### NTCP modeling

The differences in dose deposition patterns between photon and proton therapy are exploited in the Netherlands in the “model-based approach.”^269^ Here, NTCP dose-response curves are parameterized for specific therapy-related side effects, and the NTCP for each modality is compared prospectively. The results allow stratification of patients who benefit most from proton therapy in terms of reduced normal tissue effects. Similar concepts have been explored elsewhere.^270^ While a direct NTCP-driven optimization has not been applied to high LET particles, similar approaches introduced an objective based on the equivalent uniform dose^271^, ^272^ or its generalized form.^273^ Artificial inteligence-driven optimization is currently used in some general treatment planning solutions^272^ but still not used in dedicated biological dose optimization methods. A new frontier is undoubtedly in this direction.

### Modeling immunologic radiation action

Immune-modulating effects are increasingly exploited clinically in combination with immune checkpoint blockers. Model attempts are still rare, mainly because the immune system works as a complicated network of cells and signals, and their interplay with radiation burden is not sufficiently understood. While existing models are mostly tailored for photon radiation,^274^ some models consider application to particle radiation.^275^, ^276^ Particle radiation may offer enhanced effectiveness in achieving a systemic antitumoral response, including *abscopal* effects and sparing of lymphocytes, which are key players in the immune response.^277^

### Modeling spatial fractionation

Current modeling efforts focus on SFRT mechanisms related to cell-to-cell signaling and the immune system. Free radicals and reactive oxygen species are the first chemicals generated and transported between cells. Numerous studies have investigated their production and diffusion in silico.^278^, ^279^, ^280^ Experimental assays have proven the involvement of these and other later generated species (Ca^2+^, NO, exosomes, cytokines, and other proteins) in bystander-like effects,^281^, ^282^ but there are few experimental studies quantifying their production and cell survival models reflect that, for example.^283^ In van Luijk et al^284^ and Asperud et al,^285^ it was found that the inclusion of a nonlocal repair factor in their NTCP empirical model was key, supporting the idea of the immune system playing a role in SFRT. More recent analytical studies^285^ managed to model tumor volume growth by considering the activation of cytotoxic T-lymphocytes after partial and full irradiation of the tumor volume.

### Modeling FLASH effects

While the dose rate effect for protracted irradiation has been adequately described by current models,^286^ it appeared very hard to reconcile the observed protective and differential effects in the high dose rate range. Most research was concentrated on the chemical stages. Previous research in spatiotemporal effects of tracks^287^ was exploited, which contributed to determining the implausibility of some of the initially proposed hypotheses, such as transient hypoxia for oxygen depletion^26^ and the early intertrack recombination.^288^ In this context, a clear LET dependence was emphasized^289^, ^290^ and led to the hypothesis that high LET should either correlate with a strong reduction of the protective effect^15^ or several mechanisms should be discarded, focusing on others,^291^ or there is a different type of FLASH effect, specific for ions.^292^ Besides mechanistic models, a successful phenomenological description of the FLASH effect has been proposed.^293^

## Closing remarks

This review provided a panoramic overview of critical research topics and emerging new frontiers in radiobiology, highlighting the transformative innovations and techniques reshaping the field. Particle therapy should have distinct clinical advantages over conventional RT, particularly for heavy-ion and multi-ion approaches, and a robust understanding of the underlying radiobiology is critical to exploiting these benefits to enhance patient care. However, to date, we have effectively only scratched the surface of what appears possible.

We have entered a transformative era in radiobiology, one that transcends the traditional boundaries defined by physics and treatment planning verification. Historically, radiobiology was predominantly the domain of physicists, focusing primarily on the physical aspects of radiation and its application in treatment planning. This foundational work was crucial in ensuring the safety and efficacy of RT. However, we have now moved into a new era of radiobiology, where the focus has shifted toward understanding the molecular and systemic mechanisms of radiation response, and the differing effects noted on a per-ion, per-treatment, per-method basis. This new approach is not just about delivering radiation to a target; it is about comprehending the biological responses from the organism itself down to the molecular drivers elicited by different irradiation modalities.

However, for further advancements, a robust funding infrastructure is needed, beginning with a framework for simpler and faster access to particle facilities. New experimental rooms should be dedicated to radiobiology in existing PT centers, and new centers should be developed with a robust study of the underlying radiobiology in mind. Today, securing an hour or 2 of beamtime may require over 6 months of applications, paperwork, and planning. A network is necessary that allows those working in this sector to know the facilities that enable in vitro and in vivo experiments and to understand the procedures for accessing these facilities in a rapid manner. In the meantime, radiobiologists should explore new ways to perform experiments, investigating novel radiobiology model systems and allowing for detailed explorations of cellular and molecular mechanisms.

Traditional radiobiology requires advanced modeling techniques, including RBE modeling for treatment planning, NTCP modeling, and others, but we must recognize that today’s radiobiology requires understanding and as a discipline needs new and advanced models for immunologic radiation action, spatial fractionation, and FLASH effects, among others, in order to provide deeper insights into the complex interactions between radiation and biological systems.

This new era of radiobiology is characterized by a comprehensive understanding of the molecular underpinnings of RT. It is an exciting time where interdisciplinary collaboration is essential, combining the expertise of physicists, biologists, and clinicians to develop innovative treatments that are both effective and safe.

## Author Contributions

All the authors contributed equally to the writing.

## Declaration of Conflicts of Interest

The authors declare that they have no known competing financial interests or personal relationships that could have appeared to influence the work reported in this paper.
